# Role of epicardial adipose tissue in diabetic cardiomyopathy through the lens of cardiovascular magnetic resonance imaging – a narrative review

**DOI:** 10.1177/20420188241229540

**Published:** 2024-03-10

**Authors:** Sindhoora Kotha, Sven Plein, John P. Greenwood, Eylem Levelt

**Affiliations:** Department of Biomedical Imaging Science, Multidisciplinary Cardiovascular Research Centre, Leeds Institute of Cardiovascular and Metabolic Medicine, University of Leeds, Leeds, UK; Department of Cardiology, Leeds Teaching Hospitals NHS Trust, Leeds, UK; Department of Biomedical Imaging Science, Multidisciplinary Cardiovascular Research Centre, Leeds Institute of Cardiovascular and Metabolic Medicine, University of Leeds, Leeds, UK; Department of Cardiology, Leeds Teaching Hospitals NHS Trust, Leeds, UK; Department of Biomedical Imaging Science, Multidisciplinary Cardiovascular Research Centre, Leeds Institute of Cardiovascular and Metabolic Medicine, University of Leeds, Leeds, UK; Department of Cardiology, Leeds Teaching Hospitals NHS Trust, Leeds, UK; Department of Biomedical Imaging Science, Multidisciplinary Cardiovascular Research Centre, Leeds Institute of Cardiovascular and Metabolic Medicine, University of Leeds, Leeds LS2 9JT, UK; Department of Cardiology, Leeds Teaching Hospitals NHS Trust, Leeds LS1 3EX, UK

**Keywords:** cardiovascular MRI, diabetes, epicardial adipose tissue

## Abstract

Accumulating evidence suggests that ectopic/visceral adiposity may play a key role in the pathogenesis of nonischaemic cardiovascular diseases associated with type 2 diabetes. Epicardial adipose tissue (EAT) is a complex visceral fat depot, covering 80% of the cardiac surface with anatomical and functional contiguity to the myocardium and coronary arteries. EAT interacts with the biology of the underlying myocardium by secreting a wide range of adipokines. Magnetic resonance imaging (MRI) is the reference modality for structural and functional imaging of the heart. The technique is now also emerging as the reference imaging modality for EAT quantification. With this narrative review, we (a) surveyed contemporary clinical studies that utilized cardiovascular MRI to characterize EAT (studies published 2010–2023); (b) listed the clinical trials monitoring the response to treatment in EAT size as well as myocardial functional and structural parameters and (c) discussed the potential pathophysiological role of EAT in the development of diabetic cardiomyopathy. We concluded that increased EAT quantity and its inflammatory phenotype correlate with early signs of left ventricle dysfunction and may have a role in the pathogenesis of cardiac disease in diabetes with and without coronary artery disease.

## Introduction

Epicardial adipose tissue (EAT) is a complex endocrine organ that has been shown to have functions beyond thermogenesis and mechanical protection of the heart.^1–3^ EAT is part of the visceral adipose tissue (VAT), which is localized between the epicardium of the heart and the visceral layer of the pericardium. EAT surrounds approximately 80% of the cardiac surface with no fascia separating it from the myocardium and lies in close proximity to the coronary arteries.^1,4,5^

Adipose tissue is histologically classified into two types, brown adipose tissue (BAT) and white adipose tissue (WAT), which are visibly distinguishable based on tissue colour. EAT is a form of WAT but uniquely also has features of BAT, such as being composed of smaller adipocytes and expressing uncoupling protein 1 (UCP1).^1,6^ UCP1 is a mitochondrial protein dedicated to adaptive thermogenesis by stimulating high levels of fatty acid oxidation while uncoupling mitochondrial oxidation from ATP synthesis, thereby provoking energy dissipation as heat, a specialized function performed by BAT.^7,8^ Therefore, normal functioning BAT may protect against obesity and type 2 diabetes (T2D).^9^ Patients with obesity have less BAT volume and heat loss *via* UCP1.^1^ Furthermore, studies indicate that glucose uptake by BAT is reduced in T2D^10^ and there is reduced expression of peroxisome proliferator-activated receptor-gamma coactivator-1alpha (PGC-1α) in the EAT of T2D patients with coronary artery disease (CAD), which is a key regulator of energy metabolism.^6,11^

The pathophysiological role of EAT can be attributed to the several bioactive products it secretes, collectively termed ‘adipokines’ that regulate vascular tone, inflammation, endothelial function and vascular smooth muscle migration.^3,12^ EAT contains macrophages and cluster differentiation 8 cells that influence local immune function. It has been seen that in healthy lean individuals, there is a predominance of the anti-inflammatory phenotype of macrophages, which can switch to being pro-inflammatory in obesity and other metabolic conditions.^13^ Yang *et al.*^14^ demonstrated differential gene expression in people with T2D with higher expression of genes associated with inflammation and cytokines. It has also been speculated that by altering the inflammatory response and cytokine activity in EAT, diabetes predisposes patients to detrimental cardiovascular effects.^14^

EAT has dichotomous functional characteristics, both adverse and protective, interacting locally with the coronary arteries and the myocardium through paracrine and vasocrine pathways. Under physiological conditions, EAT exerts a protective effect on the coronary arteries and supplies heat to the myocardium.^15,16^ Its pathological increase and the coexistence of other metabolic and haemodynamic abnormalities turn it into an adverse lipotoxic, prothrombotic and pro-inflammatory organ.^17,18^ Under physiological conditions, processes within the EAT serve as protective forces; however, in metabolically diseased states, EAT is infiltrated by inflammatory cells, leading to translational changes within the tissue and tipping the scales towards a pro-inflammatory and pro-fibrotic phenotype of EAT.^2,19,20^

**Figure 1.**
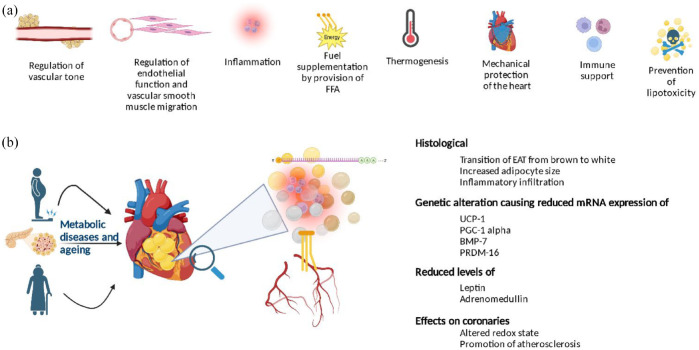
(a) Schematic representation of EAT function. (b) Intermodulation of EAT and cardiac tissue. BMP7, bone morphogenic protein-7; EAT, epicardial adipose tissue; FFA, free fatty acids; PGC-1α, peroxisome proliferator activated receptor gamma co-activator-1 alpha; PRDM-16, PR/SET domain 16; UCP-1, uncoupling protein-1.

Figure 1 summarizes the physiological and pathological role of EAT in relation to the heart.

### EAT and cardiovascular disease

Prior systematic reviews and meta-analyses have addressed the potential role of EAT in the pathophysiology of several cardiovascular conditions, including ischaemic heart disease,^21,22^ atrial fibrillation^23,24^ and heart failure with preserved ejection fraction.^25^ Patients with CAD were shown to have higher levels of reactive oxygen species in the EAT than patients without CAD.^26^ Whether this is a cause or consequence of CAD or other cardiac pathology remains to be elucidated. However, suggesting a potential causal relationship, endothelial microparticles carrying microRNAs have been shown to contribute to atherosclerosis by causing inflammation in perivascular adipose tissue, which is a form of EAT in close proximity to coronary arteries.^27^ Moreover, BAT-specific genes such as UCP-1, PGC-1α, bone morphogenetic protein 7 and PR/SET domain 16 were found to be lower in EAT from patients with CAD than in EAT from those without CAD.^26^ These findings were interpreted as a transition of brown features of EAT to white or beige features in the presence of CAD. Furthermore, adipocyte infiltration of the atrial myocardium and inflammatory cytokines from the neighbouring EAT were shown to contribute to the development of atrial fibrillation.^28,29^

### EAT in diabetes and obesity

Heart failure is the leading cardiovascular complication of diabetes. Key manifestations of myocardial disease in patients with diabetes include mitochondrial dysfunction, impaired mitochondrial calcium (Ca^2+^) handling, excess inflammation, diffuse myocardial fibrosis, cardiac hypertrophy, microangiopathy and abnormal cardiac metabolism.^30,31^ While multiple studies also suggest that EAT alterations may play a significant mechanistic role in the pathophysiology of myocardial disease in patients with diabetes, there is a paucity of systematic or narrative reviews summarizing the findings of these studies.

Several cardiovascular imaging studies have established significantly increased EAT area/volume in patients with T2D.^18,32–35^ VAT accumulation may be one of the main underlying factors for developing insulin resistance, and insulin resistance syndrome due to obesity may be the first pathological stage in the long-lasting asymptomatic period of T2D.^1,36^ Studies using invasive EAT biopsies and histological tissue assessments suggest that in metabolic disease states such as T2D and obesity, a shift between anti-inflammatory and pro-inflammatory adipocytokines within the EAT occurs, with this imbalance predisposing to chronic, low-grade inflammation, which may contribute to the development of cardiovascular disease.^14^

Moreover, the adipose tissue expandability hypothesis states that adipocytes have a maximum capacity and that over-expansion of adipocytes can lead to hypoxia and inappropriate fat storage. Hypoxia within adipose tissue, in turn, can lead to adipocyte insulin resistance through inhibition of insulin receptor tyrosine phosphorylation and decreased glucose transport.^37^ Failure to store fat effectively increases dyslipidaemia and ectopic fat deposition in tissues such as the skeletal muscle and liver, which further impairs insulin signalling in conditions such as T2D and obesity.^38^

### Imaging of EAT

**Figure 2.**
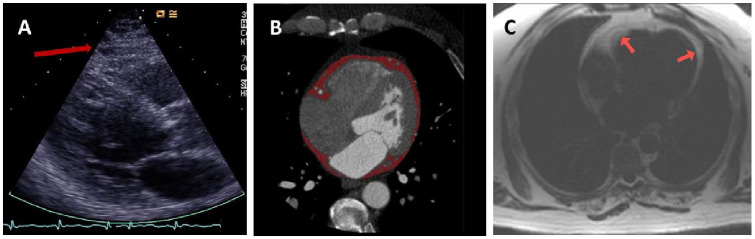
(a) Imaging of EAT by echocardiography. (b) Cardiac computed tomography^18^ (reproduced with permission). (c) Cardiovascular magnetic resonance imaging. EAT, epicardial adipose tissue.

**Figure 3.**
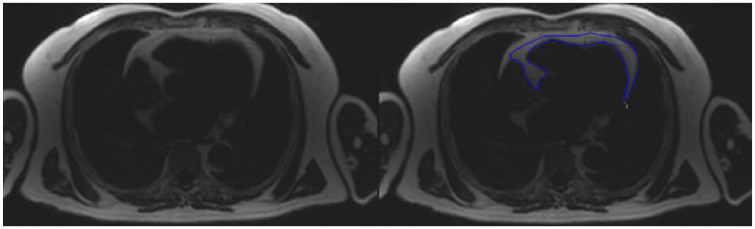
Example of manual contouring of the EAT area. A linear hypointensity enables the differentiation of EAT from pericardial adipose tissue. The area is measured with the atrioventricular junction as limits. The contours are manually drawn in all slices, and a cumulative area is expressed as the final quantity. EAT, epicardial adipose tissue.

EAT can be assessed, and different types of VAT can be differentiated using imaging techniques such as echocardiography, computed tomography (CT) and magnetic resonance imaging (MRI) (Figures 2 and 3).

Transthoracic echocardiography is an inexpensive, easily available and time-efficient modality for visualizing EAT and measuring its localized thickness.^39^ CT and cardiovascular MRI techniques have also been utilized to image EAT and offer the advantage over echocardiography of providing volumetric evaluation of EAT. Several systematic reviews have ascertained the utility of CT techniques in assessing EAT quantification and its impact on or association with the development of cardiac disease, especially CAD.^40,22^ Cardiovascular MRI is now considered the reference modality for adipose tissue evaluation, including the EAT^41^ and provides a reproducible assessment of EAT volume^42^ as well as being the best diagnostic imaging modality for reproducible assessment of cardiac volumes, mass and function.^43^ Additionally, cardiovascular MRI has been effectively used to detect subtle early-onset cardiac changes in patients with diabetes, such as alterations in myocardial strain, perfusion and myocardial lipid accumulation.^30^ The detection of subclinical dysfunction is a marker of heart failure risk and presents a potential target for reducing incident heart failure in patients with T2D.^44^ For the assessment of metabolic imaging biomarkers, magnetic resonance spectroscopy (MRS) has been utilized in many patients with diabetes. Proton (^1^H)-MRS allows for the non-invasive measurement of cardiac triglyceride content. Using this non-invasive technique, myocardial triglyceride content is increased 1.5- to 2.3-fold in patients with T2D.^45–47^ Consequently, with its clear superiority for assessment of cardiomyopathy as the reference test,^48^ its ability to detect ischaemia associated with CAD^49,50^ and an added advantage of metabolic assessment, cardiovascular MRI is an obvious one-stop shop for conveniently studying EAT characteristics and their impact on the heart. This narrative review focuses on the currently available evidence from studies that utilized cardiac MRI as the primary imaging tool in examining the role of EAT in T2D.

### MRI assessment of EAT

Various methods and pulse sequences have been used to evaluate EAT, which is usually measured either as area or volume. These MRI sequences included dark blood prepared T1-weighted multi-slice turbo spin–echo pulse sequence with a water suppression pre-pulse, 3D-mDixon sequence, standard balanced steady-state-free precession (b-SSFP) cardiovascular MRI and multi-echo gradient echo sequence to acquire water/fat images. As examples of these MRI studies, Flüchter *et al.*,^51^ in 2007, used dark blood prepared T1-weighted multi-slice turbo spin–echo pulse sequence with water suppression prepulse for acquisition and contoured EAT in end-diastolic short axis images covering the whole of left (LV) and right ventricles (RV). Homsi *et al.*^52^ assessed EAT using a 3D-mDixon sequence to acquire the images and subsequently analysed the EAT volume offline using MATLAB (MathWorks Inc., Natick, MA, USA). This method allowed volume quantification of EAT and required relatively little time per analysis. The authors suggested that this 3D-Dixon sequence could be easily integrated into any routine workflow.^52^ Furthermore, b-SSFP cardiovascular MRI was used by Leo *et al.* to evaluate EAT characteristics in detail. This sequence allowed a clear distinction of adipose tissue from myocardium, blood and other surrounding muscles.^42^ Chowdhary *et al.*^32^ used multi-echo gradient echo cardiovascular MRI acquisition to acquire water/fat images allowing measurement of EAT area with a clear definition of the adipose tissue borders.

To overcome the time-consuming nature of manual EAT segmentation, semiautomated and fully automated artificial intelligence (AI)-based programmes have been developed.^53,54^ Currently used manual methods to contour EAT may take up to 13 min by an expert cardiologist, whereas this is reduced to 5 min by automated methods.^55^

## Purpose

We aimed to close the gap in the literature of reviews summarizing the latest evidence on the utility of cardiovascular MRI in assessing the association of EAT quantification with ischaemic as well as nonischaemic myocardial disease in patients with T2D.

## Methods

### Search selection criteria and strategy

Studies investigating EAT characteristics and associations in participants with T2D were reviewed. MEDLINE was systematically searched using the keywords ‘epicardial fat’, ‘EAT’ and ‘diabetes’. Recent systematic reviews^56–60^ (covering the period of the last 5 years, between 2018 and 2023) were screened for relevant studies. The following inclusion criteria were used:

1. Studies that included participants with T2D as their primary study group or provided results specific to the T2D group.
2. Studies that utilized cardiac magnetic resonance (CMR) for quantitative measurement of EAT.
3. Studies that specifically assessed the relationship of EAT with cardiac function.

### Search results

**Figure 4.**
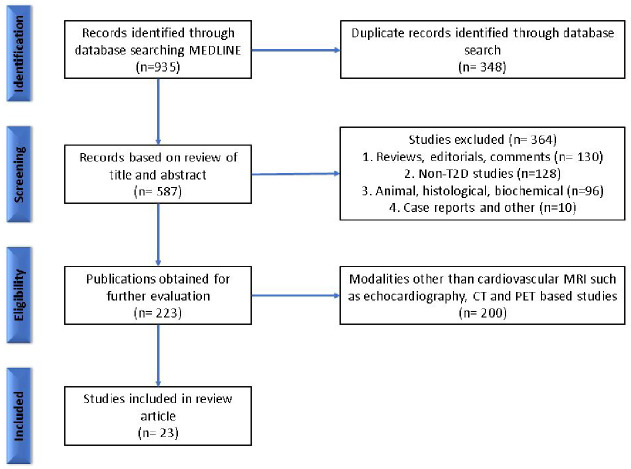
PRISMA (Preferred Reporting Items for Systematic Reviews and Meta-Analyses) flow diagram. CT, computed tomography; PET, positron emission tomography.

The study identification and selection are demonstrated in the flow diagram in Figure 4. The search was performed by a single operator (SK). The search generated 935 publications. A total of 348 duplicates were identified and excluded. Reviews, editorials, comments and responses to comments amounted to 130 publications. Eligible participants were adult patients with T2D. After reviewing the abstracts, studies that did not include patients with T2D or did not classify results for this group were excluded (*n* = 128). Studies that utilized modalities other than CMR, such as echocardiography, cardiac CT and positron emission tomography (PET), totalled 200 publications. Preclinical, histopathological and biochemical studies were excluded (*n* = 96). Eventually, 23 publications that met the inclusion criteria were included in this review (Figure 4). The description of these studies was divided into (1) observational, (2) methodological, (3) clinical trials and (4) other interventions.

Unique studies were identified by the first authors of associated publications. Patient characteristics of the studies included in this review are summarized in Tables 1 and 2. The interventional study methodologies were assessed for quality by a single operator (SK) (Supplemental Table 1).

**Table 1.** Observational studies that assessed EAT by MRI.

Authors	Journal, year	Cohort	Objective (specific to T2D group)	Results
Zhu *et al.*^61^	*Acad Radiol*, 2022	20 controls and 69 T2D patients with normal LV ejection fraction	To assess the association between EAT volume and biventricular global longitudinal strain and strain rate in patients with T2D.	EAT volume was higher in patients with T2D compared with controls. EAT measurements were associated with biventricular longitudinal systolic function and diastolic function in T2D patients.
Edin *et al.*^62^	*Front Cardiovasc Med*, 2022	46 T2D patients and 46 healthy controls	To investigate the relationship between regional fat depots and the measures of LV structure and function.	EAT volume was the only parameter associated with diastolic function when adjusting for the presence of diabetes and sex.
Chowdhary *et al.*^32^	*Obesity*, 2022	20 overweight T2D and 15 lean T2D patients	Comparisons of adipose tissue distribution, and cardiac structural and functional alterations between the asymptomatic normal weight and overweight T2D patients.	The overweight T2D group had a higher EAT area compared with the normal-weight T2D.
Huang *et al.*^35^	*J Magn Reson Imaging*, 2022	56 newly diagnosed T2D, 57 with long term T2D, 48 healthy controls	To investigate the extent of EAT volume by MRI in postmenopausal women with and without diabetes and explore the relationship between the EAT volume and microvascular function using first-pass perfusion.	EAT volume is significantly higher in T2D patients compared to normal controls. Patients with longer T2D duration had significantly higher EAT volume. Higher EAT volume and menopausal age were independently associated with myocardial microvascular dysfunction.
Haberka *et al.*^63^	*Pol Arch Intern Med*, 2019	33 T2D undergoing CABG and 33 no T2D undergoing CABG	To evaluate the link between EFV and PF volume, relative expressions of several genes in EF, PF and PVF and corresponding serum cytokines in patients with multivessel CAD in relation to DM	EFV was higher in the group with T2D. PLTP was higher in the T2D group. The T2D group showed higher expression of RAGE, FGF-21 and ADM expression in EF.
Rado *et al.*^64^	*Br J Radiol*, 2019	220 healthy controls, 100 prediabetes, 52 T2D	Associations between epicardial and paracardial fat and impaired glucose tolerance as well as LV alterations	Significant increase in epicardial and paracardial fat depots from normal glucose tolerance to prediabetes to T2D. EF showed an association with subclinical LV impairment.
Al-Talabany *et al.*^33^	*BMC Cardiovasc Disord*, 2018	145 volunteers 34 T2D and CVD; 54 T2D no CVD; 28 no T2D with CVD; 29 no T2D and no CVD	Assess the association between EAT, metabolic and inflammatory biomarkers, peripheral arterial stiffness and LV mass.	Patients with overt cardiometabolic disease have significantly more EAT than those without. EAT area is associated with aortic PWV. CD40 ligand and IL-6 levels significantly correlated with EAT and aortic PWV.
Homsi *et al.*^34^	*Acta Radiol*, 2018	156 hypertensive patients (subgrouped based on MI or no MI and T2DM or no T2DM), 20 controls	To explore the association between EAT volume, PF volume and aortic PWV in patients with treated hypertension.	A significant association between EAT volume and aortic PWV. The presence of MI is associated with higher EAT volume. In hypertension without MI, the T2D group had higher EAT volume than the patients with no T2D. EAT volume and aortic stiffness both increased in the hypertensive patients with T2D
Kim *et al.*^65^	*Cardiovasc Diabetol*, 2012	100 asymptomatic T2DM	To assess the association between EAT thickness and myocardial ischaemia, as well as coronary artery stenosis in asymptomatic T2D using CMR.	EAT thickness was an independent predictor of significant coronary artery stenosis.
Gaborit *et al.*^66^	*Int J Obes* (*Lond*), 2012	33 lean healthy volunteers, 13 morbidly obese T2D, 17 morbidly obese with no T2D	The link between EFV and MTGC and metabolic risk and compared to VAT.	EFV is higher in obese T2D compared to obese with no T2D. MTGC was not significantly different in obese with and without T2D.

ADM, adrenomedullin; CABG, coronary artery bypass graft; CAD, coronary artery disease; CD40, cluster differentiation 40; CMR, cardiac magnetic resonance; CVD, cardiovascular disease; DM, diabetes mellitus; EAT, epicardial adipose tissue; EF, epicardial fat; EFV, epicardial fat volume; FGF, fibroblast growth factor; IL-6, interleukin-6; LV, left ventricle; MI, myocardial infarction; MRI, magnetic resonance imaging; MTGC, myocardial triglyceride content; PF, paracardial fat; PVF, perivascular fat; PLTP, phospholipid transfer protein; PWV, pulsed wave velocity; RAGE, receptor for advanced glycation end products; T2DM, type 2 diabetes mellitus; VAT, visceral adipose tissue.

**Table 2.** Interventional studies.

Authors	Journal, year	Cohort	Objective (specific to T2D group)	Results
Daudé *et al.*^53^	*Diagnostics* (*Basel*), 2022	67 with T2D, 12 with obesity but no T2D, 21 healthy	EAT area on 4Ch cine MRI multi-frame images using state-of-the-art FCNs for cardiac image segmentation, that were adapted to segment EAT, PAT and cardiac ventricles	Optimized U-Net was better suited to provide EAT area estimation with a 14.2% precision for the clinically relevant upper three-quarters of the targeted EAT range
Evin *et al.*^54^	*Cardiovasc Diabetol*, 2016	19 with obesity and T2D 19 age- and gender-matched controls	A novel MRI method was used for LA strain measurement and the two-point Dixon method was used for measuring EAT. The relationship between EAT and atrial strain was assessed	LA strain is strongly correlated with BMI and epicardial fat supporting an association between adiposity and LA strain

BMI, body mass index; 4Ch, 4 chamber; EAT, epicardial adipose tissue; FCN, fully convolutional network; LA, left atrium; MRI, magnetic resonance imaging; PAT, pericardial adipose tissue; T2D, type 2 diabetes.

## Observational studies

Multiple studies have demonstrated significantly increased EAT area or volume in patients with T2D.^18,32–35,66^ Al-Talabany *et al.*^33^ demonstrated in their study of 145 patients (subdivided into four groups based on the presence of T2D and CAD) that the EAT area was significantly associated with arterial stiffness. They also showed that inflammatory markers such as cluster differentiation 40 ligand and interleukin-6 were significantly correlated with EAT volume and aortic pulse wave velocity.^33^ This was supported by Homsi *et al.*^34^ in their study of 156 patients with treated hypertension *versus* 20 controls. The patients were subdivided based on whether they had prior myocardial infarction and/or T2D. The EAT volume and aortic stiffness were significantly higher in those with T2D.^34^ Huang *et al.* conducted a study on 161 postmenopausal women, of whom 56 had newly diagnosed T2D, 57 had long-term T2D and 48 were healthy controls. In addition to the increased EAT volume in both T2D groups (newly diagnosed and chronic T2D) compared to healthy controls, they also showed that increased EAT volume was independently associated with coronary microvascular dysfunction.^35^

A retrospective, single-centre study by Kim *et al.*^65^ of 100 asymptomatic patients with T2D showed that EAT thickness was significantly higher in those with significant CAD (defined as a signal reduction or stenosis >50% on CMR angiography) than in those without. They concluded that EAT thickness was an independent predictor of significant CAD.^65^

With regard to the studies that assessed the association of EAT with LV parameters, Edin *et al*.,^62^ in their study of 46 patients with T2D and 46 matched healthy controls, showed that the impairment of diastolic function was related to higher EAT volumes. Furthermore, Zhu *et al.* demonstrated a negative correlation of EAT volume with LV global longitudinal strain (GLS), LV longitudinal strain rate, RVGLS and RV longitudinal strain rate in patients with T2D. Rado *et al.*^64^ explored the association of EAT area with subclinical LV impairment (combined endpoint based on LV concentricity index, amount of scar assessed by late gadolinium enhancement and LV ejection fraction) in a study that included patients with prediabetes, established T2D and healthy controls. They concluded that EAT area remained significantly associated with subclinical LV impairment even after adjustment for age, sex, smoking, hypertension, low-density lipoprotein, T2D and VAT.

Haberka *et al.*^63^ in a single-centre cross-sectional study that included 66 participants who were undergoing coronary artery bypass grafting surgery for multivessel CAD (33 with T2D and 33 without T2D) combined imaging and histological analysis to characterize T2D-associated changes in EAT. This study also concluded that T2D is associated with higher EAT volume on imaging. In addition, they showed reduced messenger ribonucleic acid (mRNA) expression of fibroblast growth factor 21 (cardioprotective) and increased mRNA expression of pro-adrenomedullin (cardioprotective in ischaemia/reperfusion injury) and receptor for advanced glycation end products (involved in inflammatory processes and metabolic dysfunction) in EAT.^63^

Table 1 summarizes the observational studies and includes their key objectives and results.

## Methodological studies

Two studies presented novel MRI techniques to assess EAT quantification. Daudé *et al.*^53^ employed fully convolutional networks that were used for cardiac volume segmentation techniques to measure the EAT area on horizontal long-axis four-chamber cine MRI multi-frame images. This approach was developed to overcome interobserver variability and shorten the time-consuming nature of manual analysis.^53^

Evin *et al.*^54^ developed a novel method to assess LA strain by feature tracking and used the two-point Dixon technique to measure epicardial fat. They assessed left atrial strain utilizing feature tracking. In this study, EAT and intramyocardial fat were separately measured following segmentation of the Dixon data into fat and water images. A semiautomatic method was applied for EAT quantification.^54^

Table 2 summarizes the two studies and includes their key objectives and results.

## Pharmaceutical clinical trials targeting EAT

Several interventional studies have used novel diabetes therapies for pharmacological modulation of the EAT. Overall, the evidence from the studies included in this review showed conflicting results for the effects of these drugs on the quantitative measures of EAT. Among the studies that used sodium–glucose cotransporter-2 (SGLT2) inhibitors, Gaborit *et al.*^67^ found that empagliflozin led to weight loss and reduced liver fat but had no effect on the EAT volume or myocardial fat content as measured by CMR and ^1^H-MRS, respectively. By contrast, Bouchi *et al.*^68^ in their study of 19 T2D patients found that another SGLT2 inhibitor, luseogliflozin, reduced EAT volume but had no effect on hepatic fat accumulation. They further showed that EAT volume reduction was associated with the improvement of systemic microinflammation and reduction in body weight.^68^ A reduction in EAT volume was also shown by Fukuda *et al.*^69^ in their study of nine T2D patients with increased VAT area (⩾100 cm^2^).

A further group of studies used glucagon-like peptide 1 receptor agonists (GLP1-RA). The effect of exenatide was studied by Dutour *et al*.,^70^ who showed that exenatide was an effective treatment to reduce liver fat content and EAT in patients with T2D and obesity and that these effects were mainly weight-loss dependent. A double-blind, placebo-controlled trial by van Eyk *et al.*^71^ showed that liraglutide reduced VAT, but other adipose tissue compartments, including EAT, were not changed by liraglutide treatment. Supporting this observation, another placebo-controlled randomized trial with liraglutide showed no effect on EAT volume.^72^ However, contradicting these findings, Zhao *et al*.^73^ showed a statistically significant reduction in EAT thickness after 3 months of treatment with liraglutide.

A potent PPAR-γ (peroxisome proliferator activator receptor-γ) agonist pioglitazone was another glucose-lowering drug that was studied for its effects on EAT in a small case–control study of 12 T2D patients *versus* 12 healthy controls by Moody *et al.*^74^ They demonstrated that pioglitazone reduces the EAT volume and that the reduction in EAT size is associated with an improvement in insulin sensitivity.^74^

Among the nondiabetic drugs studied, Fiore *et al.*^75^ assessed the effect of the phosphodiesterase-5 inhibitor sildenafil on EAT volume and inflammatory marker expression in 59 patients with T2D. This study found that sildenafil treatment is associated with reductions in EAT volume in T2D.^75^

Table 3 shows the list of interventional studies that aim to modify EAT.

**Table 3.** Drug interventional studies.

Authors	Journal, year	Cohort	Objective (specific to T2D group)	Results
Moody *et al.*^74^	*Diabetes Obes Metab*, 2022	12 T2D patients without manifest CVD, 12 healthy controls	To assess the effect of pioglitazone on EAT area in T2D patients and its relationship to changes in LV diastolic function.	Pioglitazone reduced EAT area and the decrease in EAT correlated with the improvements in insulin sensitivity.
Zhao *et al.*^73^	*J Diabetes Res*, 2021	21 T2D	Explore if liraglutide reduces cardiovascular events by acting on EAT in T2DM with abdominal obesity.	Liraglutide reduced EAT thickness after 3 months of treatment.
Gaborit *et al.*^67^	*Cardiovasc Diabetol*, 2021	56 T2D patients, 24 C57/BL HFHS mice model	To test if empagliflozin can modulate the ectopic fat stores and myocardial energetics in HFHS mice and T2D patients.	12 weeks of empagliflozin did not modulate the EAT volume in T2D patients. In the mice, empagliflozin did not affect the myocardial fat.
Bizino *et al.*^72^	*Diabetologia*, 2020	49 T2D BMI >25 kg/m^2^	To assess the effect of liraglutide on ectopic fat accumulation in individuals with T2D.	EAT area did not change significantly between groups.
van Eyk *et al.*^71^	*Cardiovasc Diabetol*, 2019	47 South Asian patients with T2D	To assess the effect of treatment with liraglutide for 26 weeks on ectopic fat deposition and HbA1c	Visceral adipose tissue volume was decreased by liraglutide but not by placebo. Other adipose tissue compartments were not affected by liraglutide.
Fukuda *et al.*^69^	*Diabetes Ther*, 2017	9 T2D with increased VFA (⩾100 cm^2^)	To investigate the effect of ipragliflozin on EFV in nonobese Japanese patients with T2D	Ipragliflozin significantly reduces EFV in parallel with the reduction of weight and the improvement of glycaemic control, lipid profile and insulin resistance
Bouchi *et al.*^68^	*Cardiovasc Diabetol*, 2017	19 T2D patients	To test if luseogliflozin could reduce EAT volume in Japanese patients with T2D.	Luseogliflozin significantly reduced EAT volume at 12 weeks and this was in parallel with the improvement of systemic microinflammation and reduction of body weight.
Dutour *et al.*^70^	*Diabetes Obes Metab*, 2016	44 patients with uncontrolled T2D and obesity	To investigate the effect of GLP-1 analogues on ectopic fat stores	Exenatide treatment reduced the EAT volume in T2D patients with obesity, and these effects were mainly driven by weight loss.
Fiore *et al.*^75^	*J Clin Endocrinol Metab*, 2016	59 T2D patients; 18 male BKS.Cg-Dock7^m^+/+Lepr^db^J (db/db) mice	To investigate whether PDE5i affects EAT and what mechanisms are involved, using microarray-based profiling of pharmacologically modulated micro-RNA.	Sildenafil reduced EAT volume in T2D patients after the changes in inflammatory markers such as MCP-1 were included in the model. Sildenafil regulated miR-22-3p expression in cardiomyocytes.

BKS.Cg-Dock7m+/+LeprdbJ (db/db) mice, genetically modified mice to manifest obesity, type 2 diabetes; BMI, body mass index; CVD, cardiovascular disease; EAT, epicardial adipose tissue; EFV, epicardial fat volume; GLP, glucagon-like peptide; HbA1c, glycated haemoglobin; HFHS, high fat high sugar; LV, left ventricle; MCP, monocyte chemoattractant protein; PDE5i, phosphodiesterase-5 inhibitor; T2DM, type 2 diabetes mellitus; VFA, visceral fat area.

## Nonpharmaceutical interventions targeting EAT

Nonpharmaceutical interventions targeting EAT included gastric bypass surgery and exercise training. Van Schinkel *et al.* studied the effects of bariatric surgery on ectopic adipose tissue deposition. In addition to EAT volume, they also measured paracardial fat volume, which is brown mediastinal adipose tissue located external to the parietal pericardium and contiguous with perivascular aortic adipose tissue. They found that Roux-en-Y gastric bypass surgery caused a substantial reduction in EAT volume at the 16-week follow-up and that there was a higher proportional decrease in paracardial volume as opposed to EAT volume.^76^

To investigate the effects of exercise, Jonker *et al.* conducted a prospective cohort study of 12 patients with T2D evaluating the effects of a 6-month individualized exercise training regimen. They showed that exercise reduced the paracardial fat but did not have any effect on EAT volume or intramyocardial triglyceride content.^77^

Table 4 summarizes the two interventional studies and their key objectives and results.

**Table 4.** Other interventional studies.

Authors	Journal, year	Cohort	Objective (specific to T2D group)	Results
Van Schinkel *et al.*^76^	*Clin Endocrinol* (*Oxf*), 2014	10 insulin-dependent T2D patients with obesity.	To assess pericardial fat, myocardial triglyceride content and cardiac function before and 4 months after RYGB surgery	Substantial reduction in ectopic cardiac fat was shown on a 16-week follow-up after RYGB surgery. There was a higher relative proportional decrease in PF volume as compared to EAT volume changes.
Jonker *et al.*^77^	*Radiology*, 2013	12 T2D patients	To assess the effects of an exercise intervention on organ-specific fat accumulation and cardiac function in T2D.	Exercise reduced PF but did not affect intramyocardial TG or EFV.

EAT, epicardial adipose tissue; EFV, epicardial fat volume; PF, paracardial fat; RYGB, Roux-en-Y gastric bypass; T2D, type 2 diabetes; TG, triglyceride.

## Discussion

In summary, this narrative review demonstrated that there are a significant number of contemporary studies that have focused on phenotyping EAT by MRI technology in patients with T2D, while assessments of changes in EAT quantity are not yet widely used as a surrogate endpoint for clinical trials. There are significant inconsistencies in terms of the MRI pulse sequence and analysis methodology used by the observational studies evaluating EAT characteristics, which limits the generalization of their findings and the promotion of MRI-assessed EAT quantity as an imaging biomarker for future clinical trials. Nevertheless, there is consistent evidence from imaging studies, as well as histopathological studies, that EAT may play a significant role in the diabetic cardiomyopathy process. As a result, agreement on specific standards for the MRI evaluation of the EAT quantity, such as the most reproducible pulse sequence and postprocessing technique, is required to ensure consistent quality of these assessments and validation of MRI-assessed EAT quantity as a useful imaging biomarker for multicentre large clinical studies.

## Limitations and strengths

This narrative review aimed to summarize the available evidence on the EAT phenotype in diabetic heart disease and interventional studies that target EAT size in patients with T2D. The MEDLINE electronic archive was systematically searched. The literature search terms were kept relatively broad, and the review was updated regularly to reduce the probability of omission of relevant studies. The included studies assessed sufficiently similar cohorts of patients on account of physiological or clinical characteristics and comorbid factors, making their results comparable. The included clinical trials were scrutinized for their quality (Supplemental Table 1).

While MEDLINE is a very large and regularly updated archive, we have not used EMBASE or searched for conference abstracts. Therefore, there remains a theoretical risk of omission of a small number of studies that were only included in EMBASE or recent conference papers. Although methods of a systematic review were followed, it has been presented as a narrative review as data extraction of relevant studies for the review was conducted by a single operator (SK) and prior registration of systematic review was not done.

## Conclusion and future directions

EAT quantity and metabolic characteristics have emerged as important factors implicated in the development and modulation of not only obstructive CAD but also nonischaemic diabetic cardiomyopathy. EAT quantities correlate with early signs of left ventricular dysfunction in patients with T2D. Moreover, there is evidence that genetic and molecular changes in EAT may appear before any obvious clinically apparent measure of cardiac dysfunction in this population.

Larger-scale randomized controlled trials are required to confirm the effect of SGLT2 inhibitors and GLP1-RA treatments, which have already been shown to have several other favourable effects on cardiac physiology, on EAT remodelling. Furthermore, early histological studies have suggested potential genetic and molecular targets that can potentially be modified to promote the favourable effects of EAT on the heart.

Finally, AI algorithms are exceedingly being used in often time-consuming cardiovascular imaging tasks, including image segmentation, detection of pathologies and patient selection. AI algorithms have the potential to improve the robustness of information extracted from cardiac images. AI-powered imaging data analysis has reached human-level or above human-level performance in many applications with the efficiency of modern machine learning methods such as deep learning using convolutional neural networks. While not specifically used for EAT quantification, AI technology has been implemented in the population-based UK Biobank cohort study dataset by Ardissino *et al*.^78^ for quantification of pericardial adipose tissue. Such large-scale studies are also necessary to validate and standardize EAT quantification by cardiovascular MRI to enable robust and statistically strong associations between EAT and cardiovascular health.

## Supplemental Material

sj-docx-1-tae-10.1177_20420188241229540 – Supplemental material for Role of epicardial adipose tissue in diabetic cardiomyopathy through the lens of cardiovascular magnetic resonance imaging – a narrative review Supplemental material, sj-docx-1-tae-10.1177_20420188241229540 for Role of epicardial adipose tissue in diabetic cardiomyopathy through the lens of cardiovascular magnetic resonance imaging – a narrative review by Sindhoora Kotha, Sven Plein, John P. Greenwood and Eylem Levelt in Therapeutic Advances in Endocrinology and Metabolism

## Declarations

**Ethics approval and consent to participate:** Not applicable.

**Consent for publication:** Not applicable.

**Availability of data and materials:** Data sharing is not relevant to this article, as it is a review and not original research.
