# Supplementary material for: Role of epicardial adipose tissue in diabetic cardiomyopathy through the lens of cardiovascular magnetic resonance imaging – a narrative review
Source: Ther Adv Endocrinol Metab. 2024 Mar 10;15:20420188241229540. doi: 10.1177/20420188241229540 (PMC10929063; doi:10.1177/20420188241229540)
Supplement: sj-docx-1-tae-10.1177_20420188241229540 – Supplemental material for Role of epicardial adipose tissue in diabetic cardiomyopathy through the lens of cardiovascular magnetic resonance imaging – a narrative review [file sj-docx-1-tae-10.1177_20420188241229540.docx]

# Supplementary material

| **Authors** |  | Moody et al | Zhao et al | Gaborit et al | Bizino et al | van Eyk et al | Fukuda et al | Bouchi et al | Dutour et al | Fiore et al | van Schinkel et al | Jonker et al |
| --- | --- | --- | --- | --- | --- | --- | --- | --- | --- | --- | --- | --- |
| **Study type** |  | Controlled | Single arm | RCT | RCT | RCT | Single arm | Single arm | RCT | RCT | Single arm | Single arm |
| **Blinding** |  | Open label | Open label | Double | Quadruple | Quadruple | Open label | Open label | Uncertain | Double | Multiple | Open label |
|  |  |  |  |  |  |  |  |  |  |  |  |  |
| **EAT outcome** |  | Primary | Primary | Primary | Secondary | Secondary | Primary | Primary | Primary | Primary | Primary | Primary |
| **Substudy** |  | Yes | No | No | No | No | No | No | No | Yes | No | No |
|  |  |  |  |  |  |  |  |  |  |  |  |  |
| **Selection bias** | Random sequence generation | No | N/A | Yes | Yes | Yes | N/A | N/A | Yes | Yes | N/A | N/A |
|  | Allocation concealment | No | N/A | Yes | Yes | Yes | N/A | N/A | Not specified | No | N/A | N/A |
|  | Comparable baseline | Yes | N/A | Yes | Yes | Yes | N/A | N/A | Yes | Unclear | N/A | N/A |
|  |  |  |  |  |  |  |  |  |  |  |  |  |
| **Performance bias** | All groups same care apart from intervention | Yes | N/A | Yes | Yes | Yes | N/A | N/A | Yes | Yes | Yes | Yes |
|  | Participants blinded | N/A | No | Yes | Yes | Yes | No | No | Not specified | Yes | N/A | N/A |
|  | Individuals administering care were blinded | N/A | No | Yes | Yes | Yes | No | No | Not specified | No | N/A | N/A |
|  |  |  |  |  |  |  |  |  |  |  |  |  |
| **Attrition bias** | All groups FU for equal length of time | Yes | N/A | Yes | Yes | Yes | N/A | N/A | Yes | Yes | Yes | Yes |
|  | Incomplete outcome data, which participants | No | Yes, ones that didn’t complete the study | No (ITT and per protocol analysis) | No (ITT analysis) | No (ITT and per protocol analysis) | No | No | No (ITT and per protocol analysis) | No (ITT analysis) | Unclear | No |
|  |  |  |  |  |  |  |  |  |  |  |  |  |
| **Detection bias** | Appropriate length of FU | Yes | Yes | Yes | Yes | Yes | Yes | Yes | Yes | Yes | Short | Yes |
|  | Precise definition of outcome | Yes | Yes | Yes | Yes | Yes | Yes | Yes | Yes | Yes | Yes | Yes |
|  | Reliable method to determine outcome | Yes | Yes | Yes | Yes | Yes | Yes | Yes | Yes | Yes | Yes | Yes |
|  | Investigators blinded to participants exposure to intervention | N/A | Not specified | Yes | Yes | Yes | Yes | Yes | Not specified | Yes | Not specified | N/A |

Supplementary materials table 1: Quality assessment of trials. (RCT, randomised controlled trial; N/A, not applicable; ITT, intention to treat; FU, follow-up).

| **Authors** |  | Zhu et al | Edin at al | Chowdhary et al | Huang et al | Haberka et al | Rado et al | Al-Talabany et al | Homsi et al | Kim et al | Gaborit et al |
| --- | --- | --- | --- | --- | --- | --- | --- | --- | --- | --- | --- |
| **Type of study** |  | Cross-sectional | Cross-sectional | Cross-sectional | Cross-sectional | Cross-sectional | Cross-sectional | Cross-sectional | Cross-sectional | Cross-sectional | Cross-sectional |
| **Sub type** |  | Retrospective | Retrospective | Prospective | Retrospective | Prospective | Retrospective | Prospective | Prospective | Prospective | Prospective |
|  |  | Case control | Case control | Cohort | Case control | Cohort | Case‒control | Cohort | Cohort | Cohort | Cohort |
|  | Substudy | No | No | No | No | No | No | Yes | No | Yes | Yes |
| **No. of groups** |  | 2 | 2 | 4 | 3 | 2 | 3 | 4 | 5 | 1 | 4 |
| **Centres** |  | Single | Multiple | Single | Single | Single | Single | Multiple | Not specified | Single | Not specified |
|  | Clearly, defined inclusion criteria? | Yes | Yes | Yes | Yes | Yes | Yes | Yes | No | Yes | Yes |
|  | Comparable baseline characteristics? | Yes | Yes | Yes | Yes | Yes | Yes | No | Yes | N/A | Yes |
|  | Study population and setting clearly defined? | No | Yes | Yes | Yes | Yes | Yes | Yes | No | Yes | No |
|  | Any confounding factors? | Yes | Yes | Yes | Yes | Yes | Yes | Yes | Yes | Yes | Yes |
|  | Strategies used to deal with confounding | Matching, linear regression | Restriction, matching, regression | Restriction, matching | Matching, linear regression | Restriction, matching | Restriction, matching, regression, | Restriction, Regression | Regression, restriction | Regression, restriction | Regression, matching, restriction |
|  | Reliable methods to assess exposure? | Yes | Yes | Yes | Yes | Yes | Yes | Yes | Yes | Yes | Ye |
|  | Reliable methods to assess outcomes? | Yes | Yes | Yes | Yes | Yes | Yes | Yes | Yes | Yes | Yes |
|  | Investigators blinded to participants? | Not specified | Not specified | Yes | Not specified | Not specified | Yes | Yes | Not specified | Yes | Not specified |
|  | Appropriate statistical tools used? | Yes | Yes | Yes | Yes | Yes | Yes | Yes | Yes | Yes | Yes |
|  |  |  |  |  |  |  |  |  |  |  |  |
|  |  |  |  |  |  |  |  |  |  |  |  |

Supplementary materials table 2: Quality assessment of cross-sectional studies. (N/A, not applicable).
